# Determining the effects of hyperthermia on the tumor and acute normal tissue response of FLASH radiation

**DOI:** 10.2340/1651-226X.2025.44043

**Published:** 2025-10-21

**Authors:** Priyanshu Manojkumar Sinha, Line Kristensen, Charlemagne Asonganyi Folefac, Lars Hjorth Præstegaard, Lone Hoffmann, Per Rugaard Poulsen, Michael Robert Horsman, Brita Singers Sørensen

**Affiliations:** a Experimental Clinical Oncology – Department of Oncology, Aarhus University Hospital, Aarhus, Denmark; b Danish Centre for Particle Therapy, Aarhus University Hospital, Aarhus, Denmark; c Department of Oncology, Aarhus University Hospital, Aarhus, Denmark

**Keywords:** electron radiation, conventional dose rate (CONV), ultra-high dose rate (FLASH), hyperthermia, tumor growth time, acute skin toxicity

## Abstract

**Introduction:**

There is limited indication of how hyperthermia would influence the unique proposition of FLASH radiation – its ability to maintain comparable tumor response while offering protection to normal tissues. Hence, this study was designed to investigate the potential of combining FLASH radiation with hyperthermia.

**Materials and methods:**

Experiments were performed using female CDF1 mice, where the tumor bearing or non-tumor bearing right hind legs were irradiated with either conventional dose rate (CONV) or FLASH radiation ± hyperthermia. Hyperthermia was applied 30 minutes after radiation at 42.5°C for 60 minutes. The tumor endpoint was growth delay to three times its initial treatment volume (TGT3) and the normal tissue endpoint was an acute skin toxicity of score 2.5 and above, characterized by moderate moist desquamation and partial leg deformity.

**Results:**

In tumor studies, the thermal enhancement ratio (TER) was 1.68 for FLASH radiotherapy and 1.50 for conventional (CONV) radiation. In acute skin toxicity studies, the TER was slightly lower, at 1.37 for FLASH and 1.29 for CONV. The dose modifying factor (DMF) in tumor studies was 1.12 but decreased to 1.00 when hyperthermia was added. Similarly, in acute skin toxicity studies, the DMF was initially 1.53 and dropped to 1.45 with the addition of hyperthermia.

**Interpretation:**

Hyperthermia significantly sensitized both the CONV and FLASH radiation, but the enhancement is comparable between the two different dose rate radiations in both tumors and normal tissues.

## Introduction

The biological effects of radiation on the critical organs in close proximity of the target tumor create one of the major challenges in radiotherapy. There is a constraint on how much radiation can be administered without causing significant normal tissue damage. Hence, a strong emphasis has been placed on optimizing radiation treatments such that the tumors are precisely and effectively targeted, while the damage to the surrounding normal tissue is kept to a minimum. One such approach is the application of ultra-high dose rate radiation, also known as FLASH radiation that has been investigated for its unique radiobiological proposition – it has shown significant normal tissue sparring properties in comparison to a standard dose rate radiation while maintaining the same anti-tumor effects [1–5]. The applied dose rates for FLASH radiotherapy are typically greater than 40 Gy/s, which is more than 100 times faster than the applied dose rates currently used for radiotherapy [6]. The protective phenomenon of FLASH radiotherapy may potentially transform the field of radiotherapy, as radioresistant tumors could be targeted with dose escalation without significant normal tissue toxicity.

Hyperthermia refers to heating of tumors to supraphysiological temperatures in the range of 40–45°C. This elevation can lead to profound physiological changes in the tumor such as increasing blood perfusion, and oxygenation which can greatly enhance radiation response. These complimentary anti-tumor effects of radiation and hyperthermia have been shown in several reviews [7–11], and the clinical application of radiotherapy with hyperthermia has shown improved local control without any significant normal tissue toxicity [7, 12, 13]. While there are several factors that have shown to influence hyperthermia–radiation interaction, the effect of dose rate on hyperthermic enhancement of radiotherapy remains debatable [10]. Previous preclinical studies had favored combining low-dose rate irradiation with hyperthermia [14, 15], while high-dose rate radiotherapy combined with hyperthermia had shown to be effective in a radioresistant cell line [16] or in an orthotropic brain glioma model [17]. While recent preclinical studies combining FLASH with a photothermal agent for hyperthermia primarily focused on drug delivery application of chemotherapeutic agents [18, 19], there is limited indication of how hyperthermia as therapy would influence the tumor and normal tissue response of FLASH radiotherapy. Here, we show how hyperthermia with clinically relevant temperatures and interval time influenced the normal tissue protection with radiation-induced acute damage as the endpoint.

## Materials and methods

### Murine tumor and acute skin toxicity (normal tissue) model

Female C3D2F1 mice (12–21 weeks old) were used with ages distributed evenly between the treatment groups. The mice were purchased from Janvier Labs (Le Genest-Saint-Isle, France) and acclimatized for at least 6 weeks before initiation of experiments. For the tumor experiments, the right hind leg of the mice was inoculated with 5–10 µl of finely chopped C3H mammary carcinoma (tumor material) and allowed to grow to the desired size of ~200 mm^3^ (range 141–257 mm^3^) before the initiation of radiation and hyperthermia. For the normal tissue studies, the experiments were carried out in a similar manner without the tumor inoculation. The sample size for each dose group was between 6 and 13 mice for both tumor and normal tissue experiments. All animal experiments adhered to the ARRIVE guidelines and were carried out as per the animal welfare policy of Aarhus University (http://dyrefaciliteter.au.dk) with the approval of the Danish Animal Experiments Inspectorate (License no: 2021-15-0201-01008).

Mice received CONV and FLASH irradiation with and without hyperthermia on the same days; however, investigators were not blinded during treatment delivery. As tumors reached the treatment volume (~200 mm^3^) on different days, randomization was not feasible for the tumor growth delay studies, and some selection of animals was required. In contrast, the acute skin toxicity studies were fully randomized, with mice assigned randomly to treatment groups. All treatments were conducted during the daytime to minimize potential variability due to circadian rhythm in the mice.

### Experimental method – CONV and FLASH electron beam radiation

The right hind leg of mice was irradiated by a horizontal 16 MeV electron beam using a FLASH-enabled research accelerator (Varian TrueBeam) [20]. Mice were unanesthetized for the radiation procedure and restrained in a specifically designed lucite jig with leg support to place the target leg in the radiation field. The target right hind leg was restrained with histoacrylic glue as described previously [4, 21–24]. The restrained mice were positioned on a plastic plate covered with lead and placed in a temperature-controlled water bath maintained between 24 and 26.1°C. A 1.5 cm brass block shielded the body to ensure that only the target right hind leg was exposed to the radiation. Two mice were placed on either side of the field center (leg separated by 3.5 cm) and irradiated simultaneously. Tumor-bearing (murine tumor studies) and non-tumor (murine acute skin toxicity studies) mice were irradiated identically, where the foot was kept 3 cm from the front of the waterbath, at 3.2 cm water-equivalent depth (dmax) corresponding to the dose maximum of the 16 MeV electron radiation. A uniform dose was delivered between 2 and 3 cm water column depth [25], and since the legs for both tumor and acute skin toxicity experiments were positioned within this region, the targets were assumed to have received an equivalent relative dose. The water bath was aligned with the isocentre of the gantry using a laser pointer system (SSD 100).

All radiations were given at an average conventional dose rate (CONV) of 0.16 Gy/s ± 0.02 [standard deviation] or an ultra-high average dose rate (FLASH) of 234 Gy/s ± 5.8 [standard deviation]. Both CONV and FLASH were given as pulsed beam with 200 pulses, 4 μs pulse width and either 0.0008 Gy/pulse for CONV and ~1.13 Gy/pulse for FLASH radiations. The absolute dose was determined at the mouse position of 3.2 cm water-equivalent beam depth, and 2–3 cm water column depth with a diamond detector (flashDiamond, PTW Freiburg) cross-calibrated against a plane parallel Roos chamber (PTW). Animals were treated across several experiments over a period of time; thus the absolute dose was adjusted for dose at mouse position and weekly accelerator output variations (CONV). For FLASH radiations, the dose was determined from a Bergoz ACCT current transformer signal, adjusted for the dose at the mouse position. For more details on beam characteristics and dose determination, see Kristensen et al. [25].

The resulting dose range for tumor studies was between 9.8 and 20.3 Gy and between 9.7 and 57.6 Gy for acute skin toxicity (normal tissue) studies. These doses were chosen to give optimal dose response curves. The exact radiation dose values and the number of relevant dose points could be inferred from Figures 2 (tumor studies) and 4 (normal tissue studies) respectively. All dose levels were shown for the tumor studies. In contrast, for the normal tissue studies, extreme dose levels that consistently produced either no (0%) or complete (100%) response and that lay outside the relevant dose–response region were omitted from the graph for the purpose of clarity.

### Experimental method – hyperthermia treatment

The right hind leg of the mice was placed in a separate temperature-controlled circulating water bath designed to deliver hyperthermia (model TE 623; Heto, Birkerød, Denmark). The applied temperature was 42.5°C for a duration of 60 minutes. To achieve this, the hyperthermia water bath thermostat was set 0.2°C higher in order to account for discrepancies between the applied water temperatures and the temperature around the target foot of the mouse [26, 27]. Hyperthermia was applied 30 minutes after both FLASH and CONV radiation, respectively.

### Tumor growth time and acute skin toxicity assay

For the tumor studies, tumor growth time assay was used to assess and compare the growth delay caused across different treatment groups. Different treatments were then initiated when the tumors reached the desired size (~200 mm^3^), and the volume was determined by the product of orthogonal diameters (length, width, and height) and π/6. The tumor was measured every working day until Day 90 but ended if the tumor volume reached ~1,200 mm^3^. On the days where the tumors were not measured (such as public holidays or weekends), the tumor size was estimated by the average of the measured volumes on the last and next working days.

The acute skin toxicity was assessed daily from Day 8 to Day 28 after irradiation with an established skin toxicity assay [28]. The grading severity is up to 3.5 (maximum damage) in small increments of 0.5. The non-treated left hind leg served as a control for skin damage assessment and the weight loss was monitored on a weekly basis. For mice exhibiting symptoms of acute radiation induced pain, painkillers via drinking water and commercial wet diet were provided for a period of up to 10 days.

### Data analysis

All data were analyzed with GraphPad PRISM version 10.3.1 [29]. For tumor experiments, the time taken for the tumor to reach three times its initial treatment volume (TGT3) was the endpoint of the tumor study. Dose–response plots were produced for different treatment groups, and the area under the curves (AUC) was determined for both CONV and FLASH radiation, with and without hyperthermia. Based on the AUC values, the dose modifying factor (DMF; describing the effect of dose rate on tumor response) and thermal enhancement ratio (TER; describing hyperthermic enhancement of tumor response) with 95% Confidence interval were calculated for tumor studies. For DMF, the value was determined as a ratio of AUC of FLASH radiation to AUC of the CONV radiation. For TER, the value was determined as the ratio of AUC for the combination therapy group to the radiation-only group.

The acute skin damage observed during the follow-up period was converted to binomial data (responders and non-responders) for an acute skin score of 2.5 and above. This score corresponds to moist desquamation in 50% of the irradiated area and partial foot deformity. A dose-response curve was fitted through the binomial data via logit analysis for both FLASH and CONV dose rate, with and without hyperthermia. From this, the dose required to generate an acute skin response of 2.5 and above in 50% of the treated mice (TD_50_) was determined for all four treatment groups. The DMF, that described the tissue sparring effects of FLASH, was calculated as the ratio of FLASH TD_50_ to CONV TD_50_. A score > 1 is indicative of normal tissue sparring potential of FLASH radiation [22, 30]. The effect of hyperthermia was also determined in a similar manner, where the TER was determined as the ratio of the TD_50_ for the thermoradiation group to the TD_50_ of the radiation-only group. The curves from the logit regression model were presented with data points that represent the percentage of acute skin responders for both CONV and FLASH radiation, with and without hyperthermia. To minimize the number of treated animals, some of the values for the radiation-only experiments are from a previous study [25].

## Results

**Figure 1:**
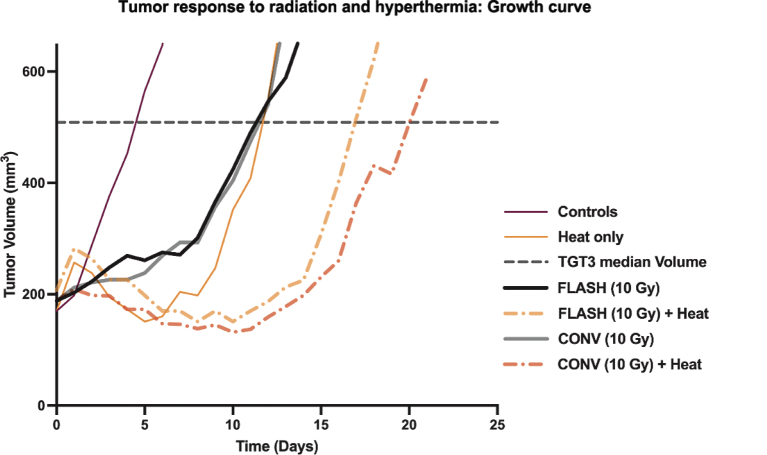
The figure shows the tumor growth curve of tumor bearing mice treated with electron beam radiation (CONV and FLASH) either alone or using radiation combined with heat. Heat (hyperthermia) was given at 42.5°C for 60 minutes and was applied 30 minutes after radiation. The figure legend, along with the applied radiation dose to get the growth curve plots is shown on the right plot. The growth curves were based on the median tumor volume measured post-treatment.

**Figure 2:**
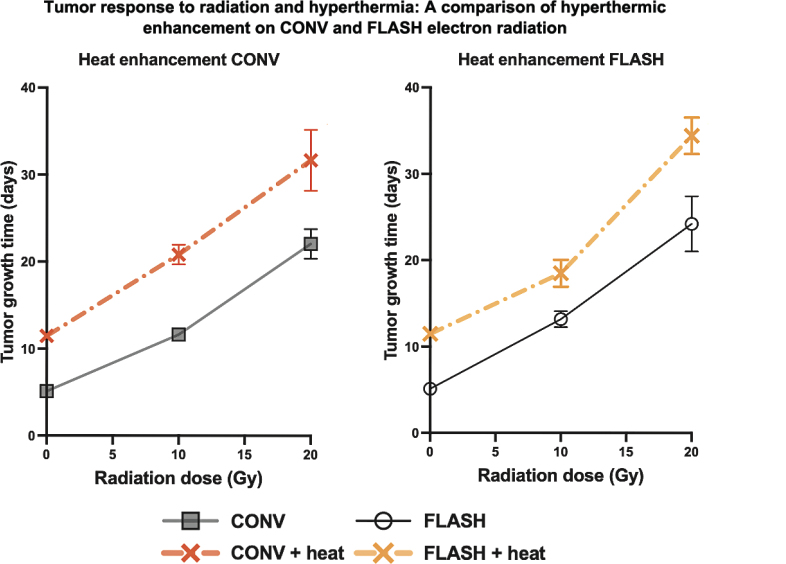
Dose response curves for electron beam radiation given with ultra-high dose rate (FLASH, black solid line & open circles) or conventional dose rate (CONV, gray solid line & squares) radiation for tumor response with TGT3 as the endpoint. Hyperthermia (42.5°C for 60 minutes, given 30 minutes after radiation) was combined with both the FLASH (dot-dashed yellow line & cross) and CONV (dot-dashed red line & cross) dose rate radiation. Each point represents the mean time for tumors to reach three times the initial treatment volume (TGT3), and the error bars represent the standard error of the mean. The results are summarized in Tables 1 and 2.

The growth of C3H mammary carcinoma tumors under various treatment conditions is illustrated in Figure 1, with the horizontal dashed line indicating the endpoint of TGT3. This is defined as the time required for each tumor to reach three times its initial volume at the time of treatment. For clarity, only a subset of treatment groups is shown in the figure; however, the TGT3 endpoint was consistently determined across all treatment groups. Both FLASH and CONV radiation resulted in a delay in tumor growth compared to controls, with further growth suppression observed when combined with hyperthermia. The most pronounced delay in reaching TGT3 was seen in the FLASH + Heat and CONV + Heat groups, indicating a thermal enhancement effect in both irradiation modalities. This is further illustrated in the dose–response curves for CONV and FLASH radiation, both alone and in combination with hyperthermia (Figure 2). When hyperthermia was added to either radiation modality, the curves shifted upward, indicating enhanced tumor growth delay, showing evidence of hyperthermia-induced radiosensitisation. Each data point represents the mean ± standard error of mean (SEM) time for tumors to reach TGT3. From these curves, the areas under the dose–response lines were calculated and used to derive the dose-modifying factor (DMF; Table 1) and the TER (Table 2). A DMF of 1.12 (95% CI: 0.73–1.73) suggests similar tumor responses between CONV and FLASH radiation alone. When combined with hyperthermia, the DMF slightly decreased to 1.00 (0.74–1.35), indicating comparable levels of hyperthermic enhancement across both modalities. This is further supported by the TER values: 1.68 (95% CI: 1.23–2.30) for CONV and 1.50 (95% CI: 0.98–2.30) for FLASH.

**Table 1:** Summary of dose-modifying factor (DMF) with and without hyperthermia (heat).

Groups	Tumor AUC (Gy*days) [95% CI]	Tumor DMF [95% CI]	Skin TD_50_ (Gy) [95% CI]	Skin DMF [95% CI]
CONV	247.47 [197.32–297.62]	**1.12** **[0.73–1.73]**	30.13 [29.10–31.20]	**1.53** **[1.46–1.61]**
FLASH	278.77 [188.72–379.82]		46.17 [44.64–47.75]	
CONV + heat	416.18 [320.69–511.68]	**1.00** **[0.74–1.35]**	23.36 [22.11–24.67]	**1.45** **[1.34–1.56]**
FLASH + heat	417.83 [337.93–497.74]		33.81 [32.17–35.58]	

Data based on Figures 2 and 4. AUC: Area under the dose response curve; TD50: dose required to induce acute skin reaction in 50% of treated mice; dDMF: dose rate Dose modifying factor: (FLASH / CONV); Significance: based on 95% CI; heat: hyperthermia at 42.5°C for 60 minutes, given 30 minutes after radiation.

**Table 2:** Summary of thermal enhancement ratio (TER) for tumor and acute skin response for conventional dose rate (CONV) and ultra-high dose rate (FLASH).

Groups	Tumor AUC (Gy*days) [95% CI]	Tumor TER [95% CI]	Skin TD_50_ (Gy) [95% CI]	Skin TER [95% CI]	TGF
CONV	247.47 [197.32–297.62]	**1.68** **[1.23–2.30]**	30.13 [29.10–31.20]	**1.29** **[1.21–1.38]**	**1.30** **[0.95–1.79]**
CONV + heat	416.18 [320.69–511.68]		23.36 [22.11–24.67]		
FLASH	278.77 [188.72–379.82]	**1.50** **[0.98–2.30]**	46.17 [44.64–47.75]	**1.37** **[1.28–1.45]**	**1.10** **[0.71–1.69]**
FLASH + heat	417.83 [337.93–497.74]		33.81 [32.17–35.58]		

Data based on Figures 2 and 4. AUC: Area under the dose response curve; TD50: Dose required to induce acute skin reaction in 50% of treated mice; TER: Thermal enhancement ratio; TGF: Therapeutic gain factor (TER tumors/TER Normal tissues); heat: hyperthermia at 42.5°C for 60 minutes, given 30 minutes after radiation.

**Figure 3:**
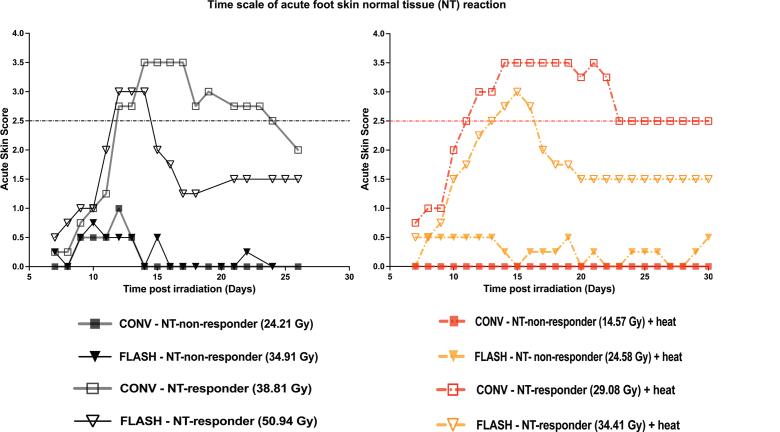
The figure shows the time scale of mice treated with electron beam radiation (FLASH [black inverted triangles] and CONV [grey squares]). Heat (hyperthermia) was given at 42.5°C for 60 minutes and was applied 30 minutes after radiation and is shown in combination with CONV [red dot-dashed] or FLASH [orange dot-dashed]. This is a representative plot of mice that were either 0% (closed symbol) or 100% (open symbol) responders for an acute skin score of ≥ 2.5 (dot-dash line). The figure legend, along with the applied radiation dose to get the time plots, is shown on the right of the growth curves.

**Figure 4:**
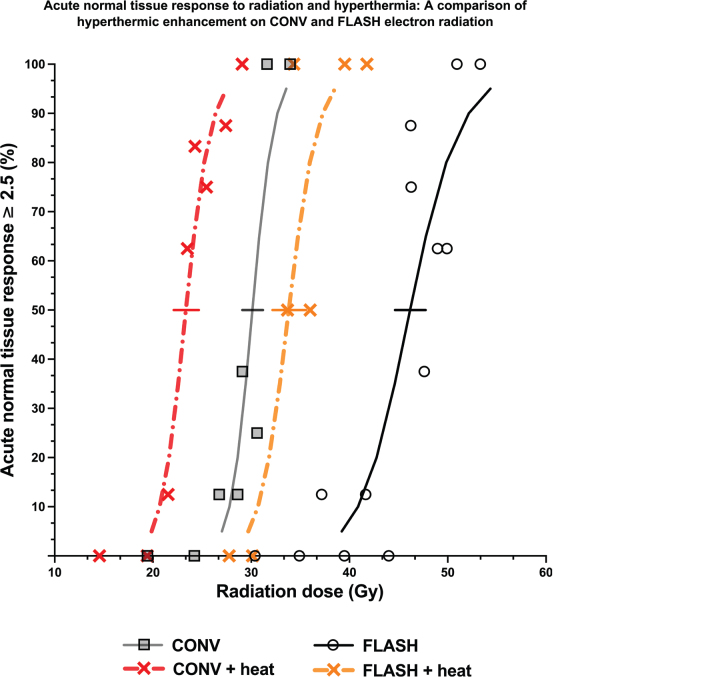
Dose response curves and the experimental points for electron beam radiation given with ultra-high dose rate (FLASH, black solid line & open circles) or conventional dose rate (CONV, grey solid line & squares) radiation for acute skin reaction of 2.5 as the endpoint. Hyperthermia (42.5°C for 60 minutes, given 30 minutes after radiation) was combined with both the FLASH (dot-dashed yellow line & cross) and CONV (dot-dashed red line & cross) dose rate radiation. Solid line at 50% represents the 95% CI of the TD50 (dose required to generate an acute skin normal tissue response [score of ≥ 2.5]) values for all treatment groups. The results are summarised in Tables 1 and 2.

The onset of acute skin response development was similar between CONV and FLASH mice, with and without hyperthermia, as seen on the time scale plot of Figure 3. The figure represents mice that were either non-responders (0%) or complete responders (100%) for acute skin score of ≥ 2.5. Responding mice reached the normal tissue endpoint around 12 days post-treatments, before the skin score came down around 18–20 days post-treatment. For the non-responding mice, the observed maximum skin reaction was rather mild, characterized by foot redness, swelling and surrounding hair loss around the skin. The acute skin toxicity that resulted in complete dose–response curves ranging from 0 to 100% responders for both CONV and FLASH dose rates with and without hyperthermia are shown in Figure 4. The FLASH dose–response curve consistently required higher radiation doses for an acute normal tissue response than CONV, irrespective of the combination with hyperthermia (42.5°C for 60 minutes). The quantified differences between all the curves presented in Figure 4 are summarized in Tables 1 and 2. They show the dose rate-DMF (Table 1) and the TER (Table 2), comparing FLASH and conventional dose rates.

As shown in Table 1, the dose-modifying factor (DMF) for the acute effects in skin without hyperthermia was 1.53 (95% CI: 1.46–1.61). With the addition of hyperthermia, the DMF decreased slightly to 1.45 (95% CI: 1.34–1.56). In Table 2, the TER for conventional (CONV) radiation for the acute effects of the skin was 1.29 (95% CI: 1.21–1.38), while for FLASH radiation it was 1.37 (95% CI: 1.28–1.45). These results provide strong evidence of considerable thermal enhancement for both CONV and FLASH electron radiation. Although hyperthermia (42.5°C for 60 minutes, administered 30 minutes after radiation) appears to enhance FLASH by approximately 8% more than CONV, the difference between the two TER values is not statistically significant due to the substantial overlap (over 50%) between their confidence intervals.

Finally, to compare tumor response and normal tissue response, therapeutic gain factor (TGF) was determined and shown in Table 2. For CONV radiation and hyperthermia, the TGF is 1.3 (0.95–1.79), while for FLASH and hyperthermia the value reduces slightly to 1.1 (0.71–1.69). When the two ratios were compared, no difference was observed, indicating comparable therapeutic benefit of combining CONV or FLASH radiation with hyperthermia.

## Discussion

Radiation and hyperthermia treatments have been shown to induce distinct biological effects that can potentially enhance tumor control while reducing damage to healthy tissues. To the best of our knowledge, previous studies regarding the dose rate effects on hyperthermic enhancement did not investigate this magnitude of dose rate difference [14, 31–33]. Hence, here we present a data supporting the combination of hyperthermia and ultra-high dose rate (FLASH) radiation, using tumor growth delay and acute skin toxicity as the endpoints.

Our tumor study results demonstrated comparable tumor growth delay between CONV and FLASH irradiation. This finding aligns with previous work using the same tumor model, where FLASH was shown not to compromise tumor response when tumor control probability was used as the endpoint [4]. In the current study, we chose tumor growth delay as the endpoint to cross-validate both tumor growth delay and tumor control probability endpoints within the same model, providing a robust evaluation of treatment efficacy. Combining hyperthermia with either CONV or FLASH radiation resulted in significant and comparable thermal enhancement. This suggests that, under the conditions where hyperthermia was 30 minutes post-irradiation, the large difference in dose rate between FLASH and CONV was not a critical factor in modulating the hyperthermic effect. Clinically, hyperthermia can be administered before or after irradiations, although the latter is more widely applied [8, 11]. In our study, hyperthermia was administered 30 minutes post-irradiation, rather than concurrently, to better reflect this clinically feasible treatment sequencing and also to primarily focus on the effects of hyperthermia on post-irradiation tumor damage response.

Next for our acute toxicity (normal tissue) studies, the mouse foot skin assay was used. This is an established model for the study of the radiation effects, where early skin reactions are scored according to a well-defined scale [28]. The acute skin damage in the current investigation was assessed for up to 30 days after treatment. This acute reaction typically begins with the onset of mild symptoms like skin erythema, alopecia and foot oedema that progressively becomes worse over time [34]. In all of the treated mice in our investigation, the onset of observed acute skin reaction followed the same pattern described earlier (Figure 3), and was independent of dose, dose rate, or if hyperthermia was applied. For the majority of the mice that reached an acute skin reaction score of 2.5 and above, the observed acute reaction was reversible, with complete disappearance of normal tissue reaction. This resolution of acute normal tissue response is largely dependent on the applied radiation dose [34]. Any residual skin reaction seen around later time points (> 20 days) was characterized by foot oedema (swelling) and alopecia (hair loss around the irradiated skin), and in some cases with foot deformity. The normal tissue endpoint applied in our study is moist desquamation, which results from epidermal cell death and subsequent reduction in epidermal cellularity and deformed skin cell morphology. This results in reduced number of cell layers, leading to reduced epidermal thickness [34].

The TGF, which represents the ratio of thermal enhancement in tumor tissue relative to normal tissue, provides a useful metric for evaluating the overall clinical benefit of combining hyperthermia with radiation. A TGF greater than 1 indicates that the addition of hyperthermia enhances tumor response more than it exacerbates normal tissue toxicity. In this study, both CONV and FLASH radiation combined with hyperthermia yielded TGFs above 1, more specifically, 1.3 for CONV and 1.1 for FLASH. This suggests a net therapeutic advantage in both cases. However, the broad and overlapping confidence intervals [CONV: 0.95–1.79; FLASH: 0.71–1.69] indicate no statistically significant difference between the two modalities. This suggests that the therapeutic benefit of hyperthermia is comparable when used with either CONV or FLASH radiation. More importantly, this indicates that adding hyperthermia does not compromise the normal tissue-sparing characteristics of FLASH and could potentially be a viable combinatorial strategy to enhance tumor response without increasing toxicity. In fact, a study by Leavitt et al. demonstrated that FLASH irradiation led to significantly greater tumor growth delay and improved survival outcomes in hypoxic (clamped) tumors compared to CONV radiation, suggesting a unique advantage of FLASH in low-oxygen environments over CONV radiation [35]. Since hyperthermia is also known to exert direct cytotoxic effects on hypoxic cells [8, 36], its combination with FLASH could potentially offer a better synergistic benefit in overcoming the radioresistance associated with tumors that have a poor oxygenation status. These insights highlight the need for further investigation, particularly in well-established hypoxic tumor models, and validate the therapeutic potential. Future studies should also evaluate different hyperthermia parameters such as temperature, timing, and duration to better understand how these variables influence treatment efficacy, as only one temperature (42.5°C) and time interval (30 minutes post-irradiation) were investigated. Such research will be essential for optimizing combination strategies and translating them into meaningful clinical applications.

## Data Availability

Data presented in this study are available from the corresponding author on reasonable request.
